# Crystal structural basis for Rv0315, an immunostimulatory antigen and inactive beta-1,3-glucanase of *Mycobacterium tuberculosis*

**DOI:** 10.1038/srep15073

**Published:** 2015-10-15

**Authors:** Wanyu Dong, Junhua Huang, Yanan Li, Yubei Tan, Zhou Shen, Yunfeng Song, Dang Wang, Shaobo Xiao, Huanchun Chen, Zhen F. Fu, Guiqing Peng

**Affiliations:** 1 The National Key Laboratory of Agricultural Microbiology, Huazhong Agricultural University, Wuhan, Hubei, China; 2 College of Veterinary Medicine, Huazhong Agricultural University, Wuhan, Hubei, China; 3 Departments of Pathology, College of Veterinary Medicine, University of Georgia, Athens, GA 30602, USA

## Abstract

*Mycobacterium tuberculosis (Mtb)* remains a leading cause of morbidity and mortality worldwide, as two billion people are latently infected with *Mtb*. To address *Mtb* drug resistance and the limitations of current vaccines, the characteristics of candidate *Mtb* vaccines need to be explored. Here, we report the three-dimensional structure of Rv0315 at 1.70 Å resolution, a novel immunostimulatory antigen of *Mtb*, and demonstrate that Rv0315 is an inactive β-1,3-glucanase of the glycoside hydrolase 16 (GH16) family. Our study further elaborates the molecular basis for the lack of glucan recognition by Rv0315. Rv0315 has a large open groove, and this particular topology cannot bind oligosaccharide chains in solution, thus explaining the lack of detectable hydrolytic activity towards its substrate. Additionally, we identified Glu-176, a conserved catalytic residue in GH16 endo-β-1,3-glucanases, as essential for Rv0315 to induce immunological responses. These results indicate that Rv0315 likely diverged from a broad-specificity ancestral GH16 glucanase, and this inactive member of the GH16 family offers new insights into the GH16 glucanase. Together, our findings suggest that an inactive β-1,3-glucanase in *Mtb* drives T-helper 1 (Th1) immune responses, which may help develop more effective vaccines against *Mtb* infection.

*Mycobacterium tuberculosis* (*Mtb*) is a leading cause of human mortality, responsible for approximately two million deaths each year. The bacterium that causes tuberculosis (TB) is able to elude the host immune system and cause disease after infection or remain latent for long periods of time^1^. These characteristics may be explained in part by many factors that were elucidated after genome sequencing of the *Mtb* H37Rv strain^2^. The H37Rv genome sequence also revealed the presence of an endo-β-1, 3-glucanase (Rv0315). Rv0315 exhibits the highest sequence similarity to GH16 family endo-β-1,3-glucanases. Rv0315 is a secreted protein of *Mtb*, and the signal peptide is cleaved by a signal peptidase before the mature protein is released^3^. Although Rv0315 is known to be secreted by *Mtb*, little is known about its function.

β-1,3-glucanases, which are classified as exo-β-1,3-glucanases (β-1,3-glucan glucohydrolase (EC 3.2.1.58)) and endo-β-1,3-glucanases (β-1,3-glucan glucano-hydrolase (EC 3.2.1.6 and EC 3.2.1.39)), are widely distributed among bacteria, fungi, and higher plants. β-1,3-glucanases have several potential applications in biotechnology, including utilization in the production of yeast extract^4^,^5^, and the use of soluble β-1,3-glucans as immune activators^6^. In plants, β-1,3-glucanases are thought to be involved in cell differentiation and defense against fungal pathogens through their ability to hydrolyze β-1,3-glucan, a major cell wall component^7^. In viruses, β-1,3-glucanases are involved in degrading the host cell wall during virus release and/or are packaged in the viral particles and thus involved in virus entry^8^. In bacteria, endo-β-1,3-glucanases and endo-β-1,3-1,4-glucanases are involved in metabolism^9^. These enzymes are closely related and catalyze the hydrolysis of polysaccharides^10^,^11^. β-1,3-glucanases act on 1,3-β-glucosyl linkages, requiring a region of unsubstituted, contiguous 1,3-β-linked glucosyl residues. In contrast, β-1,3-1,4-glucanases merely hydrolyze 1,4-β-glucosyl linkages when the glucosyl residue itself is linked at the O-3 position^12^. Endo-β-1,3-glucanases are also able to hydrolyze mixed-linkage β-1,3-1,4-glucans, such as lichenan, but prefer β-1,3-glucans, such as laminarin^13^. Despite these functional differences, most bacterial endo-β-1,3-glucanases (laminarinases) known to date share sequence similarity with endo-β-1,3-1,4-glucanases (lichenases) and belong to the GH16 family, except for a laminaripentaose-producing β-1,3-glucanase from *Streptomyces matensis*, which belongs to GH64^14^,^15^,^16^. Most bacterial laminarinases have been classified as GH16 family members, sharing a β-jelly roll fold and catalyzing the glycosyl hydrolysis reaction in a retaining mechanism. The laminarinase ZgLamA from *Zobellia galactanivorans* (ZgLamA_GH16_, PDB code 4BOW) is highly specific for the degradation of laminarin. Two conserved glutamates in ZgLamA_GH16_ (Glu-269 and Glu-274) function as the catalytic residues. *Zobellia galactanivorans* is a marine bacterium and has proven to be able to live on laminarin as sole carbon source^17^.

Laminarin, a small branched β -1,3-glucan, is the carbon storage polysaccharide of brown algae^18^. This glucan consists of two homologous series of molecules (G-series and M-series), and both series have a mean degree of polymerization of 25 glucosyl residues^19^. β-1,3-glucans, which constitute the cell walls of plants and fungi, have biological activities in immune modulation^20^,^21^,^22^. Rv0315 can induce dendritic cell (DC) maturation and activation via the activation of MAPK and NF-κB signaling, and it participates in adaptive immunity by inducing T cell immune responses toward T-helper 1 (Th1) polarization via antigen-presenting DCs^23^.

In this study, we demonstrate that Rv0315 is an inactive β-1,3-glucanase via different approaches. The biological function of Rv0315 and the molecular mechanisms underlying the activation of DCs are also investigated and discussed.

## Results

### Molecular structure of Rv0315

**Figure 1:**
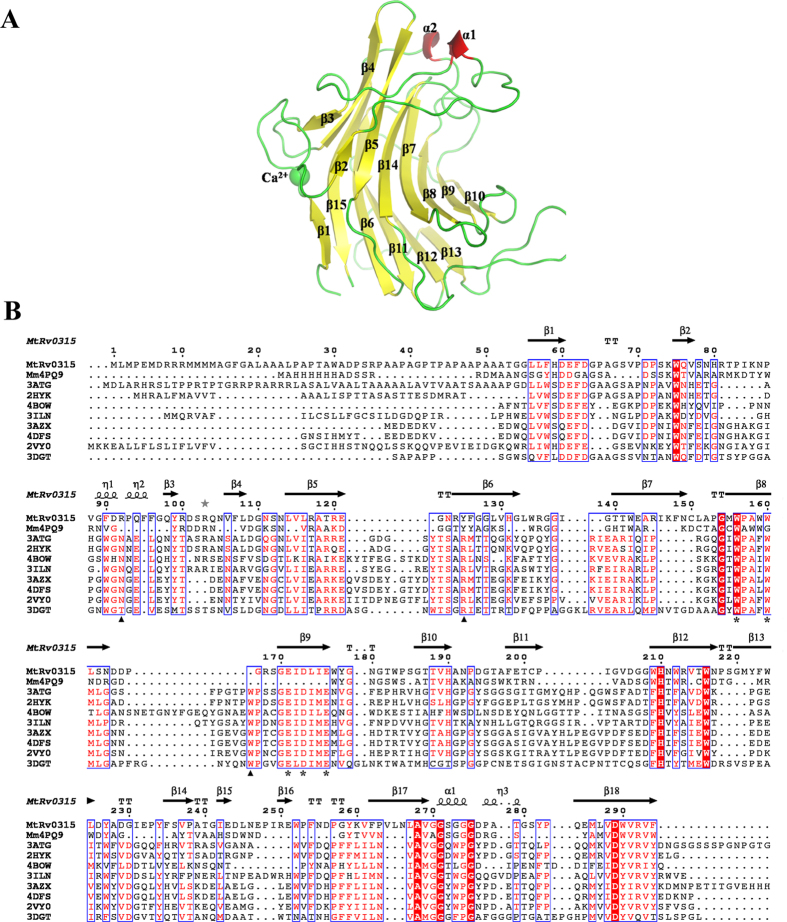
Crystal structure of Rv0315 and structure-based sequence alignment of GH16 family β-1,3-glucanases. (**A**), Overall structure of Rv0315. The β-sheets of Rv0315 are colored yellow, the α-helices are colored red, and the other parts of Rv0315 are colored green. (**B**), Rv0315 is compared with all of the GH16 laminarinases that have been structurally characterized so far. The α-helices and β-strands are shown as helices and arrows, respectively, and β-turns are marked with TT. The following sequences from the Protein Data Bank were used to create the sequence alignment: BglF from *Nocardiopsis sp. F96* (2HYK), LamR from *R. marinus* (3ILN), endo-1,3-β-glucanase from *Cellulosimicrobium cellulans* (3ATG), LamA from *Pyrococcus furiosus* (2VY0), Tpet_0899 from *Thermotoga petrophila* (4DFS), TM_0024 from *Thermotoga maritima* (3AZX), endo-1,3-β-glucanase from *Streptomyces sioyaensis* (3DGT), ZgLamA from *Z. Galactanivorans* (4BOW) and β-1,3-glucanase from *Mycobacterium Marinum* (4PQ9). Dark shaded boxes indicate invariant positions, and light shaded boxes show positions with similar residues. The amino acid residues of 4BOW related to subsites −1 and −2 are marked with an asterisk and a black triangle, respectively, below the 3DGT sequence.

Based on the genome sequence of the *Mtb* H37Rv strain, the Rv0315 gene was predicted to produce a putative β-1,3-glucanase. To determine whether Rv0315 is a β-1,3-glucanase of the GH16 family and also to facilitate crystallization assays, the nucleotide sequence of Rv0315 was cloned into the vector pET42b. Soluble recombinant protein was produced in *E. coli* BL21 (DE3) cells. A nickel ion affinity chromatography step followed by gel filtration chromatography was performed to purify Rv0315. The gel filtration chromatography analysis suggested that Rv0315 is a monomer in solution. The crystal structure of Rv0315 was solved at a resolution of 1.7 Å by molecular replacement using the structure of β-1,3-glucanase from *Mycobacterium marinum* (PDB code 4PQ9) as a search model. The crystal is monoclinic (C121), and its asymmetric unit contains two chains of Rv0315 (designated as chains A and B), and a total of 681 water molecules. Each monomer is composed of 241 amino acids (54–294). The final model of Rv0315 has been refined to *R*_*work*_ of 18.4% and *R*_*free*_ of 21.4% (Table 1). The overall structure of Rv0315 reveals that the protein adopts a sandwich-like β-jelly-roll fold, formed by the face-to-face packing of two antiparallel sheets, which is typical of GH16 enzymes. The overall structure comprises 16 β-strands forming two twisted β-sheets, two short a-helices and one calcium ion (Fig. 1A,B). The calcium ion binding site is well conserved in GH16 enzymes^24^ and has been suggested to be important for protein stability^25^,^26^,^27^. The β-sheets twist and bend, forming a substrate binding concavity. The amino acid sequence of Rv0315 is highly conserved among the different strains of *Mtb* (Fig. S1), and Rv0315 from the H37Rv strain exhibits the highest similarity to the GH16 family endo-β-1,3-glucanases (Fig. 1B).

### Rv0315 is an inactive β-1,3-glucanase

**Figure 2:**
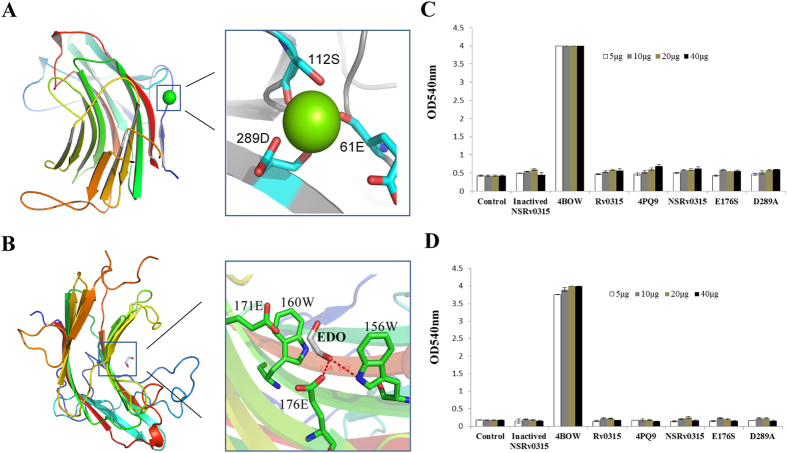
Hydrolysis of laminarin and barley β-glucan by Rv0315 or other proteins was monitored by the DNS method. (**A**) Calcium ion-binding site of Rv0315. (**B**) Substrate-binding site of Rv0315. (**C**,**D**) Experiments were performed at 37 °C in 100 mM sodium phosphate buffer at pH 7.5 with different concentrations of purified enzyme and 1.5% (w/v) laminarin (**C**) or 1% (w/v) barley β-glucan (**D**).

Rv0315 shows sequential and structural similarities to GH16 family endo-β-1,3-glucanases, a family of proteins that display β-1,3-glucanase activity. β-1,3-glucanase exhibits an endo-like degradation behavior (“endo” meaning that the enzyme attacks anywhere along the polysaccharide chain) based on an analysis of the oligosaccharide reaction products. To determine whether Rv0315 is in fact a GH16 β-1,3-glucanase, the polysaccharide-hydrolyzing function of Rv0315 was explored. Two types of polysaccharides were tested: 1,3-β-glucan (laminarin from *Laminaria digitata* (1.5% w/v)) and 1,3–1,4-β-glucan (β-D-glucan from barley (1% w/v)). The digested products were specifically analyzed by the DNS method at OD540. Rv0315 displayed no detectable activity against either polysaccharide even when the amount of the enzyme was increased (Fig. 2C,D). Rv0315 also did not show detectable activity against laminarin in the following buffers (at 100 mM) with pH values varying from 4 to 9 by increments of 0.5 pH units: phosphate citrate (pH 4–6), MOPS (pH 6–7.5), Tris-HCl (pH 7.5–8.5), and glycine-NaOH (pH 8.5–9) (data not shown).

**Figure 3:**
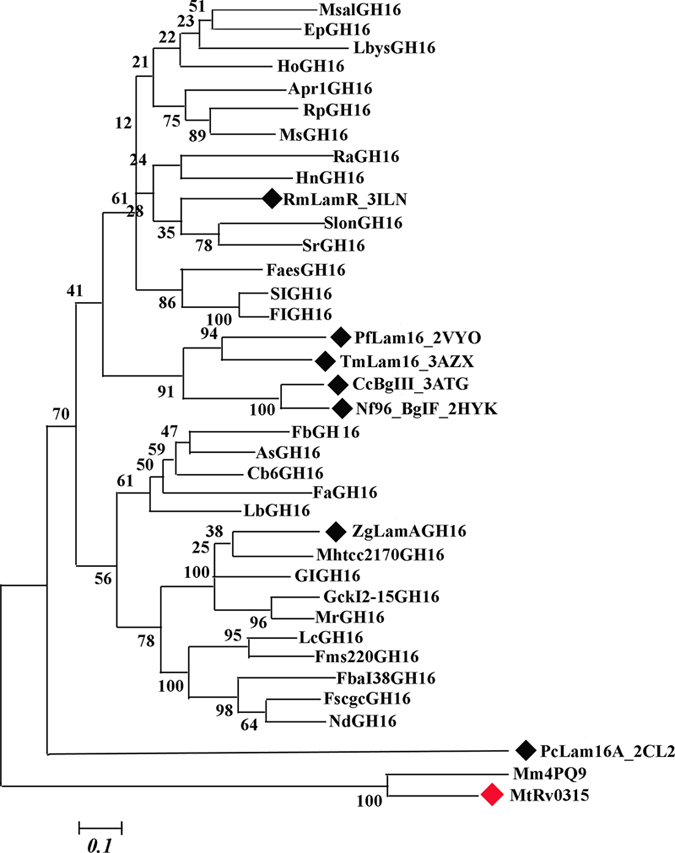
Unrooted phylogenetic tree of the Rv0315 homologues. The phylogenetic tree was derived using the neighbor-joining approach with the program MEGA5.1 (31). Numbers indicate the bootstrap values in the neighbor-joining analysis. Bootstrap values were calculated from 1,000 replicates of the alignment. The sequences correspond to characterized β-glucanases marked by black diamonds. The sequences listed below are used to form the phylogenetic tree (Marked as label, GenBank TM accession number): MtRv0315, NP_214829.1; MsalGH16, WP_010663693; EpGH16, WP_018472769; LbGH16, WP_009779440; LbysGH16, YP_003996482.1; HoGH16, YP_002508023; Apr1GH16, WP_008202826.1; RpGH16, WP_019597521; MsGH16, WP_008629242; RaGH16, WP_017930206; HnGH16, WP_022826080; RmLamR_3ILN, AAC69707; SlonGH16, WP_022834864.1; SrGH16, YP_003572933; FaesGH16, YP_007322050; SlGH16, YP_003386191.1; FlGH16, WP_009280395; FaGH16, CDF79586; FbGH16, YP_004843635; AsGH16, WP_016194601 ; Cb6GH16, WP_022831977.1; PfLam16_2VY0, AAC25554; TmLam16_3AZX, AAD35118; CcBglII_3ATG, AAC38290; Nf96_BglF_2HYK, BAE54302; ZgLamAGH16_4BOW, CAZ96583; Mhtcc2170GH16, YP_003862726; Gck-I2–15GH16, WP_008992711; MrGH16, YP_004787302; LcGH16, WP_020536539 ; Fms220GH16, WP_017842933; Fbal38GH16, WP_008253474;FscgcGH16, WP_020081599; NdGH16, YP_007376876; GlGH16, WP_006989208; PcLam16A_2CL2, BAC67687; and Mm4PQ9, 4PQ9_B.

The NSRv0315 (no-signal-peptide Rv0315) protein is composed of residues 32–294 excluding the signal peptide^3^. Two conserved glutamates in the pattern EXDX(X)E are catalytic residues^28^ in the GH16 family proteins; in Rv0315, Glu-171 is equivalent to the catalytic nucleophile, whereas Glu-176 is the general acid-base catalyst (Fig. 1B). In particular, Glu-176 forms hydrogen bonds with ethylene glycol and is a key amino acid involved in the hydrolysis reaction (Fig. 2B). Glu-176 was successfully replaced by a serine (Rv0315E176S), and Asp-289, which interacts with calcium ions (Fig. 2A) was replaced by an alanine (Rv0315D289A) by site-directed mutagenesis. Soluble mutants of Rv0315 and NSRv0315 were also produced in *E. Coli* BL21 (DE3) and purified with the same procedure. We also analyzed the phylogenetic tree of Rv0315 homologues. As shown in Fig. 3, β-1,3-glucanase from *Mycobacterium marinum* (Mm4PQ9) and Rv0315 from *Mtb* (MtbRv0315) were in a clade independent of Rv0315 homologues. All of these proteins were also assayed on laminarin and β-D-glucan from barley via a reducing sugar assay, as was done for Rv0315. However, no enzymatic activity was detected even with increasing amounts of the protein.

### Crystal structure comparison between Rv0315 and ZgLam_AGH16_

**Figure 4:**
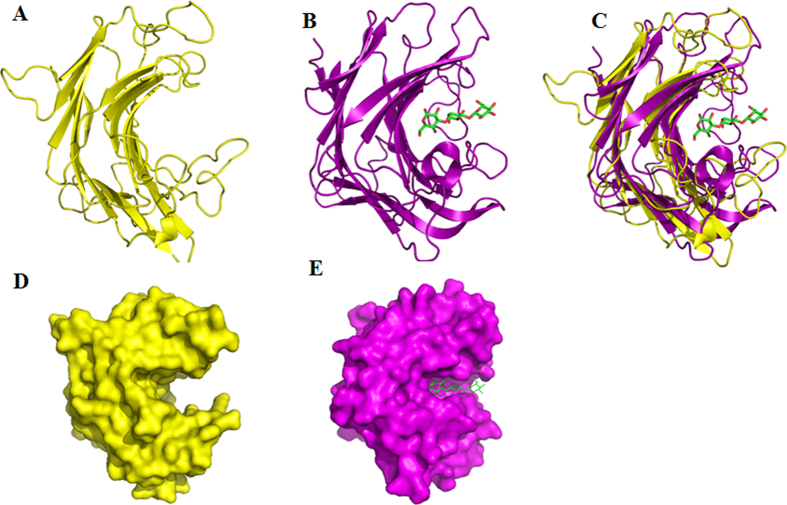
Rv0315 has a large cavity and cannot adapt to bind to oligosaccharides. (**A**,**B**) are fold representation of Rv0315 and ZgLamA_GH16_ in complex with a laminarin tetrasaccharide, respectively. (**C**) ZgLamA_GH16_ and Rv0315 are superimposed and plotted in purple and yellow, respectively. (**D**,**E**) are stereo view of the surface of Rv0315 and ZgLamA_GH16_ in complex with a laminarin tetrasaccharide, respectively. The orientations of Rv0315 are identical in (**A**,**D**), and the orientations of ZgLamA_GH16_ are identical in (**B**,**E**).

To determine why Rv0315 displays no detectable hydrolytic activity, the structures of Rv0315 and the ZgLamA_GH16_ complex were compared. Rv0315 was specifically co-crystallized with laminarin. However, only ethylene glycol molecules were observed in the structure, as seen in the wild-type protein. One of the ethylene glycol molecules was bound to subsite −1 of the catalytic cleft of each monomer, mimicking a bound glucose moiety, and the hydroxyl group O1 formed a strong hydrogen bond to Glu-176 O2 (2.7 Å). Rv0315 has a larger cavity (Fig. 4A,D) than that of the ZgLamA_GH16_ structure (Fig. 4B,E); this large cavity is not present in other known structures of the GH16 family laminarinases. Other than the different size of the cavity, the overall structure of Rv0315 was similar to that of ZgLamA_GH16_ (Fig. 4C). Structural comparison between Rv0315 and ZgLamA_GH16_ suggests that Rv0315 may lack detectable hydrolytic activity because of its wider cavity.

### Comparison of Rv0315 with other β-glucanase complexes: molecular basis of lacking laminarin recognition

**Figure 5:**
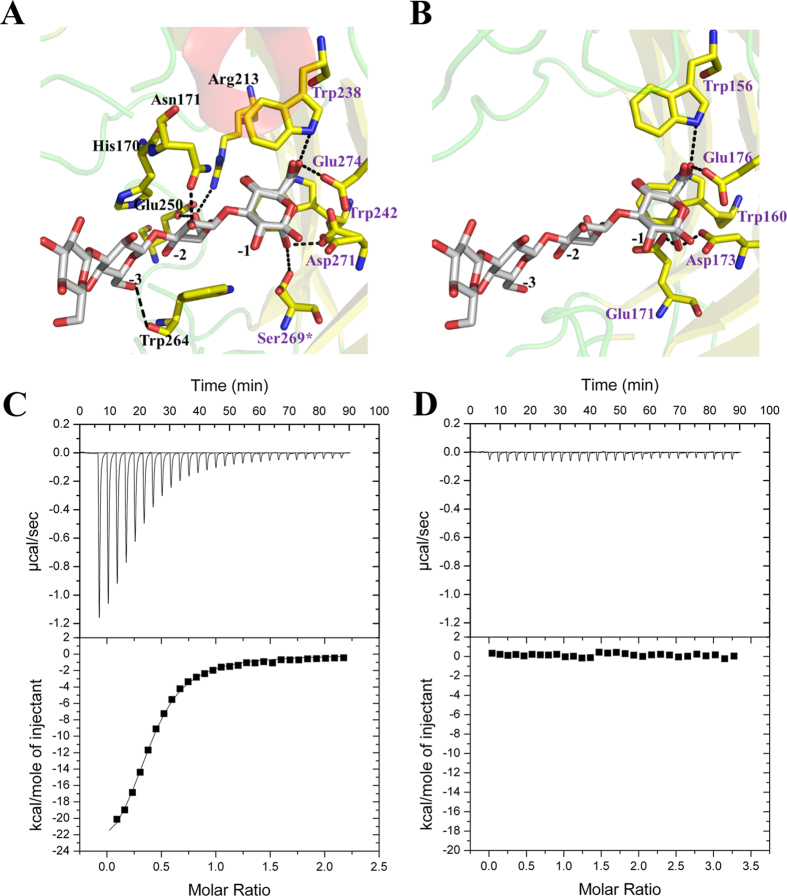
Molecular basis of the failure of Rv0315 to recognize laminarin. Structural comparison of the laminarinase ZgLamA_GH16–E269S_ (**A**) bound to laminaritetraose and superposition of laminaritetraose from ZgLamA_GH16–E269S_ and Rv0315 (**B**). The laminaritetraose is shown as gray sticks. Hydrogen bonds are depicted as dotted lines. The amino acids are colored as follows: purple indicates the residues involved in substrate recognition that are conserved between the two proteins; black indicates the residues that differ in the recognition pattern. The asterisk refers to the mutation of Glu-269 into Ser-269 in ZgLamA_GH16–E269S_. (**C**,**D**), representative ITC experiments for the titration of laminarin into (left to right) ZgLamA_GH16–E269S_ and Rv0315. At the concentrations of laminarin used, clear binding was observed for ZgLamA_GH16–E269S_ but not for Rv0315. The resulting stoichiometry (N-value) is ~0.5, *K*_*d*_ of 1.78 ± 0.0795 μM, *ΔH* of −2.684 × 10^4^ ± 551.9 cal mol-1, ΔS of −60.3 cal K-1 mol-1.

The asymmetric unit contained two Rv0315 molecules. Each protein cavity bound an ethylene glycol that formed hydrogen bonds with Glu-176 and Trp-156 as well as the additional residues Asp-173 and Glu-171. The ethylene glycol also formed hydrophobic interactions with Trp-156 and Trp-160 (Fig. 2B), which are conserved in all GH16 family enzymes and constitute a hydrophobic platform for correctly orienting the sugar ring at the −1 subsite^24^. The subsite −1 of GH16 family laminarinases is well conserved (Fig. 1B). The tryptophan residues Trp-238 and Trp-242 of ZgLamA_GH16–E269S_ were oriented in the same direction as Trp-156 and Trp-160 (Fig. 5A,B). The subsite −2 of ZgLamA_GH16–E269S_ was characterized by hydrogen bonds formed by the glucose unit and three polar residues (Asn-171, Glu-250, and Arg-213). The glucose unit at subsite −2 was sandwiched by Trp-264 and His-170. The glucose at subsite −3 was stabilized by a hydrogen bond between its hydroxyl group Glc3 O6 and the carbonyl of Trp-264 (Fig. 5A). All of these residues related to subsites −2 and −3 were not conserved in Rv0315 and could not form any direct interactions with the substrate (Figs 5B and 1B). The larger cavity of Rv0315 is also due to the lack of these residues. Given all of these differences, polysaccharides may not be able to firmly bind to the cleft of Rv0315 because of its large cavity and the lack of key residues to fix the β-glucans, leading to a lack of detectable catalytic activity for Rv0315.

The binding of polysaccharides to Rv0315 was also studied by ITC. ZgLamA_GH16_ exhibits hydrolytic activity against laminarin. The heat changes generated by titrating laminarin into ZgLamA_GH16_ may include both binding and hydrolysis. To eliminate the effect of the heat changes of hydrolysis, the inactive mutant ZgLamA_GH16–E269S_ was used as a positive control. Titrating laminarin into Rv0315 did not result in significant heat changes (Fig. 5D), whereas the titration of laminarin into ZgLamA_GH16–E269S_ resulted in relatively large exothermic heat changes (*ΔH* = −2.684 × 10^4^ ± 551.9 cal mol^−1^) and exhibited typical binding characteristics (Fig. 5C). The data obtained from the binding reactions were fitted using one set of sites binding model with a calculated stoichiometry (*N*) of ~1:2 (laminarin:ZgLamA_GH16–E269S_), indicating that one molecule of the laminarin interacts with two molecules of ZgLamA_GH16–E269S_. The *K*_*d*_ of laminarin to ZgLamA_GH16–E269S_ was determined to be 1.78 ± 0.0795 μM. All the data above suggest that laminarin cannot bind to Rv0315, resulting in a loss of enzymatic activity, consistent with the results obtained in the DNS experiment (Fig. 2C,D).

### Key Rv0315 residues involved in NF-κB pathway activation

**Figure 6:**
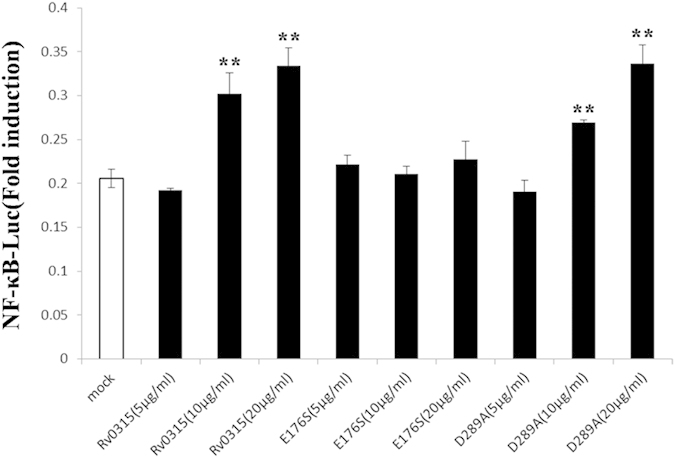
The Rv0315 and Rv0315D289A proteins activate the NF- κB signaling pathway in a dose-dependent manner. HeLa cells were co-transfected with an NF-κB reporter plasmid and pRL-TK. After 24 h, the HeLa cells were cultured for another 24 h in the presence of 5, 10, or 20 μg/ml Rv0315 protein or Rv0315 mutants and analyzed for the expression of luciferase. Mock, untreated control. TNF-α, the positive control, is not shown in the chart because the activation ratio was beyond of the limit of detection. Each value of *p < 0.05, **p < 0.01, or ***p < 0.001 was considered to be statistically significant versus mock.

Recent studies have shown that Rv0315 induces DC maturation and activation through NF-κB and MAPK signaling and also participates in adaptive immunity by inducing T cell immune responses toward Th1 polarization via antigen-presentation, which triggers T cell activation and proliferation^23^. We speculate that, driven by evolution, Rv0315 may lose its hydrolytic activity against β-glucan but still play a role in adaptive immunity. To identify the key residues of Rv0315 associated with the immune response, we generated serine or alanine mutations of calcium ion-binding (Fig. 2A) or catalytic residues (Figs 2B and 5B), respectively. These Rv0315 mutants were expressed and purified in *E. coli* BL21 cells. All of the mutant proteins showed the same expression levels, solubility, and chromatographic behaviors as the wild-type protein did. The endotoxin content in the Rv0315 and Rv0315 mutants preparations was also measured by a limulus amebocyte lysate (LAL) assay and was lower than 15 pg/ml (<0.1 UE/ml). At concentrations lower than 20 μg/ml, our recombinant protein was not cytotoxic to HeLa cells, as demonstrated by a CellTiter 96 AQueous One Solution Cell Proliferation Assay (Promega). We performed luciferase reporter assays using Rv0315 and the mutant proteins. Specifically, HeLa cells were co-transfected with a NF-κB-Luc luciferase reporter plasmid and the internal-control plasmid pRL-TK and were then treated with Rv0315 or the Rv0315 mutants. A single serine substitution for Glu-176 (Rv0315E176S) significantly decreased the activation of the NF-κB signaling pathway (Fig. 6), whereas another substitution (Rv0315D289A) had no effect. The NF-κB signaling pathway was activated by Rv0315 and Rv0315D289A in a dose-dependent manner. These mutagenesis studies suggest that Glu-176 is a key residue in the activation of the NF-κB signaling pathway and the modulation of T cell-mediated immunity by Rv0315.

### Rv0315E176S cannot induce DC maturation

**Figure 7:**
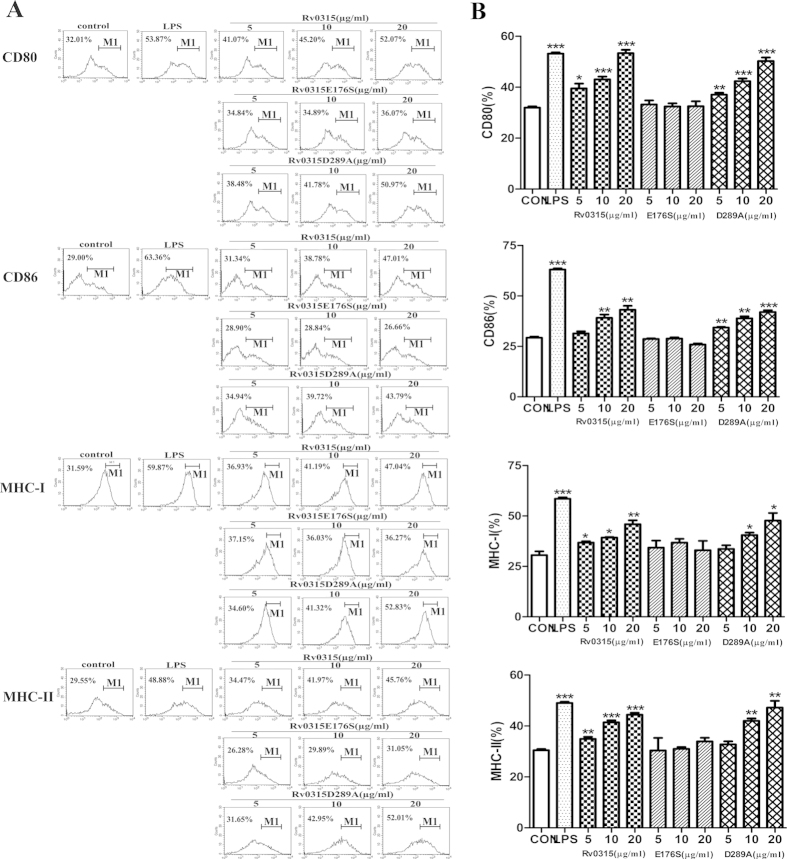
Rv0315 and Rv0315D289A induce the expression of co-stimulatory and MHC class molecules on DCs in a dose-dependent manner. DCs were cultured for 24 h in the presence of 5, 10 or 20 μg/ml Rv0315 protein or mutants and analyzed for the expression of surface markers by three-color flow cytometry. The cells were gated to select CD11c+ cells. Medium, untreated control; LPS, positive control (100 ng/ml LPS). DCs were stained with anti-CD80, anti-CD86, anti-MHC class I, or anti-MHC class II antibodies. Bar graphs show the mean ± SEM of the percentage of each surface molecule on CD11c+ cells from three independent experiments. Each value of *p < 0.05, **p < 0.01, or ***p < 0.001 was considered to be statistically significant versus untreated control.

Because NF-κB and MAPKs are essential for DC maturation, we examined whether the Rv0315E176S mutation affects the maturation of sentinel DCs to effector DCs. For this purpose, BMDCs were cultured for 6 days in RPMI 1640 medium supplemented with GM-CSF and IL-4 under standard conditions, followed by culture in the presence of 5, 10 or 20 μg/ml Rv0315 or Rv0315 mutants. LPS (200 ng/ml) was used as a positive control. The recombinant proteins were not cytotoxic to DCs at a concentration of 20 μg/ml, as determined by flow cytometry (data not shown). The expression of various cell-surface markers on DCs was analyzed by flow cytometry to identify the resulting populations of DCs. As shown in Fig. 7A,B, DCs treated with Rv0315 or the Rv0315D289A mutant presented increased expression of surface markers, including CD80, CD86, MHC class I and II. Rv0315 and Rv0315D289A each induced significant up-regulation of co-stimulatory and MHC molecules in a concentration-dependent manner. However, replacement of Glu-176 with serine (Rv0315E176S) failed to induce DC maturation.

## Discussion

Bacteria produce endo-β-1,3-glucanases (EC 3.2.1.39) and endo-β-1,3–1,4-glucanases (EC 3.2.1.73), which are closely related and have a high degree of specificity and homogeneity^13^. Endo-β-1,3–1,4-glucanases hydrolyze β-1,4-glucosyl linkages, such as lichenan, only when the glucosyl residue itself is linked at the O-3 position^12^. In contrast, endo-β-1,3-glucanases primarily catalyze the hydrolysis of glucans with 1,3-β-linkages, such as laminarin. Several biotechnological applications have been considered for endo-β-1,3-glucanases and endo-β-1,3–1,4-glucanases^29^. In the current study, we characterized the structure of Rv0315, a putative β-glucanase found in the *Mtb* genome. Based on structural and amino acid sequence alignments, Rv0315 exhibits the highest similarity to GH16 endo-β-1,3-glucanases (Fig. 1), a family of proteins that display β-1,3-glucanase activity, with two conserved glutamates playing the role of the catalytic residues^28^. However, our enzymatic activity assay showed that Rv0315 is actually an inactive β-glucanase that is ineffective for the degradation of 1,3-β-glucans, such as laminarin, and has no activity toward 1,3–1,4-β-glucans, such as β-D-glucan from barley (Fig. 2C,D). Intriguingly, ZgLamA_GH16_, which is relatively similar to Rv0315 (56% identity), is highly efficient for the degradation of laminarin (a 1,3-β-glucan) and has a residual activity towards MLG (a 1,3–1,4-β-glucan)^17^.

Structural comparisons between Rv0315 and ZgLamA_GH16_ yielded a clear explanation of how such a remarkable difference in hydrolytic activity developed between two closely related enzymes. We have also highlighted other crucial differences as the molecular basis as to why laminarin is not recognized by Rv0315 by comparing Rv0315 substrate complexes with other β-glucanase complexes. The mode of substrate recognition is partially shared between Rv0315 and ZgLamA_GH16_, but it is limited to subsite −1 (Fig. 5A,B). Subsite −2 of ZgLamA_GH16_ is characterized by hydrogen bonding between the glucose unit and three polar residues (Asn-171, Glu-250 and Arg-213), and Glu-250 belongs to an additional loop that is unique to ZgLamA_GH16_. This additional loop appears to block the negative subsites and enable ZgLamAGH16 to efficiently bind and degrade the helical-shaped laminarin^17^. Trp-264 and His-170 in ZgLamA_GH16_ form a sandwich to stabilize the glucose unit at subsite −2. The glucose at subsite −3 of ZgLamA_GH16_ is also stabilized by Trp-264, the carbonyl of which forms a hydrogen bond with the hydroxyl group Glc3 O6. The residues related to subsite −2 (Asn-171, Arg-213 and Trp-264) in ZgLamA_GH16_ are conserved in the GH16 family laminarinases (Fig. 1B). However, the Trp-264 residue of ZgLamA_GH16_ is absent in Rv0315, and Asn-171 and Arg-213 are not conserved in Rv0315. Taking all of these differences into account, presently the only known molecule that can bind Rv0315 is ethylene glycol (Fig. 2B). Polysaccharides cannot bind firmly to the catalytic cleft of Rv0315 because Rv0315 lacks key substrate-binding residues, resulting in a large open groove and enzymatic inactivity. We performed ITC experiments to further confirm that laminarin is not capable of binding to Rv0315 (Fig. 5D). Laminarin bound ZgLamA_GH16–E269S_ with a Kd value of 1.78 ± 0.0795 μM, and the resulting stoichiometry (N-value) is 1:2 (laminarin:ZgLamA_GH16–E269S_) (Fig. 5C). Laminarin consists of a G-series containing 22 ~ 28 glucosyl residues and an M-series containing 20–30 glucosyl residues. Both series have a mean degree of polymerization (dp) of 25 glycosyl residues^19^. Thus, the size of laminarin from *Laminaria digitata* is heterogeneous^19^, and its molecular weight is not an accurate value. In addition, at least four glucosyl residues can bind to ZgLamAGH16-E269S in the structure of the ZgLamA_GH16–E269S_-laminaritetraose complex. Based on the orientation of the laminarin tetrasaccharide in ZgLamA_GH16–E269S_ (Fig. 4B,E) and the characteristics of laminarin, it is possible that one molecule of laminarin can combine with two molecules of ZgLamA_GH16–E269S_.

Prolonged treatment with constant medical oversight can lead to the increased emergence of multidrug-resistant strains of *Mtb* and contributes to the difficulties in preventing and treating *Mtb* bacterial infection^30^,^31^. Furthermore, co-infection with HIV has complicated the threat as most of the anti-retroviral drugs used to treat HIV are not compatible with certain TB treatments^32^,^33^,^34^. The attenuated live *Mycobacterium bovis* vaccine Bacillus Calmette-Guerin (BCG) has been used for TB prevention^35^, but reports of its efficacy in the prevention of pulmonary TB in adults are inconsistent^36^,^37^. Therefore, new strategies for controlling TB will require the development of novel TB vaccines or the improvement of current TB vaccines. T cell-mediated immune responses are essential for the effective control of *Mtb* infection^38^,^39^, so a key requirement for generating protective immunity against TB is to activate T cells following vaccination. Byun *et al.* indicated that *Mtb* Rv0315 is an immuno-stimulatory antigen that prompts DC maturation in a concentration-dependent manner and that can induce host-favored immune responses through activation of NF-κB and MAPK signaling^23^. It is essential to decipher the functional characteristics of such mycobacterial antigens that are critical for eliciting protective immune responses. In the present study, we showed for the first time that Rv0315 is an inactive β-1,3-glucanase of *Mtb* and that Glu-176 is a key amino acid of Rv0315 whose substitution with serine interferes with substrate recognition and catalysis and significantly decreases the activation of the NF-κB signaling pathway, ultimately leading to a failure to induce DC maturation. In contrast, although Asp-289 is responsible for binding to calcium ions, its substitution with Ala has no effect on the activation of NF-κB or on DC maturation (Figs 6 and 7). Therefore, we propose that the ancestral β-1,3-glucanase in the GH16 family has broad specificity for laminarin and MLG^17^ and that Rv0315 lose its hydrolytic activity towards β-glucan but still plays a role in adaptive immunity. The loss of hydrolytic activity may thus be a recent evolutionary event. A phylogenetic analysis of the close homologues of Rv0315 and ZgLamA_GH16_ with β-1,3-glucanase further supports this hypothesis. Indeed, Rv0315 forms a solid clade with β-1,3-glucanase from *Mycobacterium marinum* (Mn4PQ9) (Fig. 3). However, the function of β-1,3-glucanase from *Mycobacterium marinum* is still unknown. The β-1,3-glucanase from *Mycobacterium marinum* is also inefficient for the degradation of laminarin and β-D-glucan from barley, similar to Rv0315 (Fig. 2C,D). In addition, none of the residues of ZgLamA_GH16_ related to subsites −2 and −3 were conserved in β-1,3-glucanase from *Mycobacterium marinum* (Fig. 1B). The observation of a clade comprising both Rv0315 and β-1,3-glucanase from *Mycobacterium marinum* gives novel insights into the evolution of this complex family of GH16 laminarinases. Although the large cavity in Rv0315 prevents the strong binding of small molecules, such as oligosaccharides, it may still interact with some macromolecules associated with the immune system. This large cavity is also more likely to be exposed to and targeted by the immune system, which may result in effects on the immune response to TB through the binding of immune molecules to Rv0315. However, the identity of the immune-related molecules that bind to Rv0315 and their detailed mechanisms remain to be determined.

It is not clear why Rv0315 became an inactive β-1,3-glucanase and lost detectable activity toward β-glucan over the course of evolution, but we propose that it would make no sense to consider Rv0315 as a β-1,3-glucanase of *Mtb*. In bacteria, β-1,3-glucanases participate in the degradation of polysaccharides utilized as a source of nutrients^40^,^41^. Our results show that Rv0315 has no hydrolytic activity (Fig. 2C,D), which is consistent with the unusual capacity of *Mtb* to catabolize host cholesterol as a source of carbon and energy, thereby sustaining a persistent infection^42^. Thus, *Mtb* can reproduce in cells with a low density of polysaccharides, and hydrolytic activity may be not necessary. By interacting with the host cells, especially macrophages, the secreted proteins of *Mtb* mediate important biological functions and are thus believed to be potentially important for pathogenesis and virulence^43^,^44^,^45^,^46^. Rv0315 is identified as a secreted protein^47^ and is a non-essential gene for the growth of *Mtb* H37Rv *in vitro*^48^. Whether Rv0315 plays a role in virulence, pathogenesis and/or the *in vivo* growth of *Mtb* needs to be further explored.

The structural and functional studies presented here provide insights into the structure-function relationship of Rv0315. In particular, we have reported the three-dimensional structure of *Mtb* Rv0315 and shown that *Mtb* Rv0315 has no hydrolytic activity against glucan polymers and thus is a non-essential gene for the optimal growth of *Mtb*^49^. Additionally, the E176 residue of Rv0315 is a key conserved catalytic residue in GH16 endo-β-1,3-glucanases and is essential for Rv0315 to initiate DC maturation. DCs are vital for the regulation of cellular immune responses during *Mtb* infection^50^, and it is well documented that cell-mediated immune responses are essential for protection against TB. Therefore, Rv0315 could be an excellent immune activator to enhance the immunogenicity of candidate *Mtb* vaccines.

## Materials and Methods

Unless otherwise indicated, all chemicals were purchased from Sigma-Aldrich.

### Bacterial strains, plasmids and growth conditions

*E. coli* DH5a and BL21 (DE3) strains (Novagene) were routinely used for the cloning and expression of recombinant genes. pET42b (Novagene) was used as a cloning and expression vector. IPTG was added at a final concentration of 0.5 mM to induce gene expression.

### Cloning and site-directed mutagenesis of Rv0315

Briefly, primers (with *NdeI* and *NotI* restriction sites in boldface) were designed to amplify the coding region by PCR from *Mtb* genomic DNA. The forward primer 5′-ggaattc**catatg**cttatgcctgagatggatc-3′ and reverse primer 5′-ccg**ctcgag**aaagacgcgcacccagtc-3′ were used. The purified PCR product was ligated to the expression vector pET42b, resulting in a recombinant protein with fused C-terminal 8X his tag (plasmid pET42b-Rv0315). Plasmid pET42b-Rv0315 was used as a template for site-directed mutagenesis, followed by splicing by overlap extension PCR^51^, and to construct NSRv0315. The two residues Glu-176 and Asp-289 were replaced by serine and alanine, respectively. The two mutagenesis primers were as follows. Mutant E176S: forward primer 5′-cgaaatcgacctgatcagttggtatggcaacgggac-3′, reverse primer 5′-gtcccgttgccataccaactgatcaggtcgatttcg-3′; mutant D289A: forward primer 5′-ggaattcCATATGcttatgcctgagatggatc-3′, reverse primer 5′-ccgCTCGAGaaagacgcgcacccatgcgacgagcatctcct-3′. The two primers (with *NdeI* and *NotI* restriction sites in boldface) of NSRv0315 were as follows. Forward primer 5′-ggaattcc**catatg**gacccgtcccggccggcc-3′, reverse primer 5′-ccg**ctcgag**aaagacgcgcacccagtc-3′). All of the mutated genes were sequenced to confirm that only the intended mutations had occurred.

### Overexpression and purification of pET42b-Rv0315 and mutants

An overnight culture of *E. coli* BL21 (DE3) pLysE harboring the expression vector pET42b-Rv0315 was harvested and resuspended in PBS. The cells were lysed by passage through an AH-1500 homogenizer (ATS Engineering Inc.) to obtain the periplasmic proteins. The recombinant protein was loaded onto a 5-ml His Trap^TM^ HP column (GE Healthcare) that was pre-equilibrated with buffer A (20 mM Tris, pH 7.4, 500 mM NaCl) and was then eluted with a linear gradient between buffer A and buffer B (20 mM Tris, pH 7.4, 500 mM NaCl, 500 mM imidazole). Fractions that contained a protein band with the desired molecular weight (34 kDa) were pooled and loaded onto a 120-ml Superdex 75 (GE Healthcare) column. Finally, the protein was eluted with buffer C (20 mM Tris, pH 7.4, 200 mM NaCl). The expression and purification of mutant proteins were identical to the procedures used for wild-type protein. pET42b-Rv0315, pET42b-NSRv0315, pET42b-Rv0315E176S and pET42b-Rv0315D289A were concentrated to 20, 7.3, 12.55 and 7.73 mg/ml, respectively, using an Amicon Ultra (10000 MWCO) centrifugal filter device (Millipore). All chromatographic steps were conducted at 4 °C. β-1,3-glucanase from *Mycobacterium marinum*, ZgLamA_GH16_ and the mutant ZgLamA_GH16–E269S_, were purified using the same procedure.

### Crystallization, data collection, structure determination, and refinement

Crystallization was performed via sitting drop vapor diffusion at 20 °C by mixing 1 μl of the protein sample and 1 μl of the reservoir solution. The initial crystallization conditions for Rv0315 were manually optimized, and single crystals were obtained as follows: 1 μl of protein at 14 mg/ml was mixed with 1 μl of reservoir solution containing 0.2 M ammonium phosphate monobasic and 20% PEG 3350. All crystals grew within 1 week in sitting drops at 20 °C and belong to the space group C121. The three-dimensional protein structure of Rv0315 was solved by molecular replacement using Phaser software^52^ with the structure of β-1,3-glucanase from *Mycobacterium marinum* as a search model. The data set was collected at the Shanghai Synchrotron Radiation Facility (SSRF) beam line BL17U1 (wavelength = 0.97910 Å, temperature = 100 K). All data sets were indexed, integrated, and scaled using HKL-3000^53^. The program package COOT^54^ was used for model building and adjusting residues. Refinement was performed with Refmac software^55^ (Table 1) . Water molecules were added using CCP4/wARP and manually inspected^56^. Structure figures were produced using PyMOL^57^.

### Sequence and structure comparison

Rv0315 homologues from the GH16 family of laminarinases with known three-dimensional crystal structures were selected^25^, and their amino acid sequences were recovered from the PDB. Multiple sequence alignments were performed using CLUSTALW2 with secondary structure depiction; colored figures were generated by ESPript^58^ based on the superimposition of the structures of the different laminarinases. The crystal structure of Rv0315 was compared to that of ZgLamA_GH16_ using COOT and PyMOL. Close homologues of Rv0315 were selected based on a BlastP search on GenBank NR for phylogenetic analysis and aligned using CLUSTALW2. The phylogenetic tree was derived from the refined alignment using the neighbor-joining method in the program MEGA 5.1^59^. The reliability of the neighbor-joining tree was estimated by bootstrap analysis with 1,000 replicates.

### Enzymatic activity assays on β-glucans

The hydrolytic activities of purified Rv0315 and the mutants were determined by measuring the amount of reducing sugars released from different β-glucans using the 3,5-dinitrosalicylic acid (DNS) method^60^. Laminarin from *Laminaria digitata*, a 1,3-β-linked glucan, has a large number of reducing ends; thus, it was reduced prior to use. Meanwhile, β-D-glucan from barley is a 1,3–1,4-β-linked glucan. The standard activity assay was carried out at 37 °C for 30 min using 1.5% (w/v) laminarin or 1% β-D-glucan from barley in 100 mM sodium phosphate buffer (pH 7.5). The reaction was stopped by the addition of 100 μl of DNS solution and by boiling at 100 °C for 7 min. The mixture was then cooled to room temperature, and the absorbance was measured at 540 nm. NS-Rv0315 was inactivated by boiling for 30 min at 100 °C as a negative control, and ZgLamA_GH16_, a β-glucanase highly specific for laminarin^17^, was used as a positive control. Substrate blanks were also included. All experiments were performed in triplicate. Protein concentrations were determined using the BCA Protein Assay Kit (Beyotime, China), with bovine serum albumin as the standard, according to the manual’s instructions.

### Isothermal titration calorimetry (ITC)

ITC experiments were performed using a VP-ITC calorimeter (GE Healthcare). Purified Rv0315 and ZgLamA_GH16–E269S_ were dialyzed against 20 mM Tris pH 7.4 200 mM NaCl overnight at 4 °C, and the tested ligand (laminarin) was dissolved in the same buffer. Twenty nine injections (10 μl each) of laminarin (200 μM, syringe) were then titrated into Rv0315 (20.7 μM, sample cell) and ZgLamA_GH16–E269S_ (19.94 μM, sample cell), respectively, at 37 °C, with 180-s intervals between injections. The control was laminarin titrated into dialysis buffer to determine the heat changes associated with diluting the ligand. The final heat changes were analyzed using the Microcal Origin 7.0 software package.

### Luciferase reporter gene assay

HeLa cells were seeded in 48-well plates and co-transfected with the NF-κB reporter plasmid at 0.05 μg/well together with the plasmid pRL-TK (Promega) at 0.01 μg/well. The pRL-TK plasmid expresses Renilla luciferase constitutively as an internal control to normalize transfection efficiency. A total of 24 h after the initial transfection, the cells were incubated with increasing concentrations (5, 10, or 20 μg/ml per well) of different proteins (Rv0315, Rv0315E176S or Rv0315D289A) for another 24 h at 37 °C, and cell lysates were then prepared. Firefly luciferase and Renilla luciferase activities were monitored by the Dual-Luciferase Reporter Assay System (Promega), according to the manufacturer’s instructions. Cells not treated with protein were used as negative controls, and cells treated with TNF-α (40 ng/ml) were used as positive controls. The data are shown as the relative firefly luciferase activity normalized to the Renilla luciferase activity and are presented as the mean value from three independent experiments.

### Generation and culture of DCs

DCs were generated from murine bone marrow monocyte cells^61^. Briefly, bone marrow was flushed from the tibiae and femurs of 6- to 8-week-old female C57BL/6 mice, treated with red blood cell lysing buffer and washed with RPMI-1640 medium. The cells were plated in 12-well culture plates (5 × 10^5^ cells/ml; 2 ml/well) using RPMI 1640 supplemented with 10% heat-inactivated FBS, 100 U/ml penicillin, 100 mg/ml streptomycin, 10 ng/ml recombinant mouse GM-CSF, and 5 ng/ml recombinant mouse IL-4 and cultured at 37 °C in the presence of 5% CO_2_. On day 3 of culture, floating cells were gently removed and fresh medium added. On day 6 or 7 of the culture, non-adherent cells and loosely adhering proliferating DC aggregates were harvested for analysis or stimulation. On day 7, over 80% of the non-adherent cells expressed CD11c.

### Flow cytometry determination of DC stimulation by wild-type and mutant Rv0315

Isolated DCs were cultured for 24 h in the presence of Rv0315 or mutant protein (5, 10 or 20 μg/ml). These BMDCs were harvested, washed with phosphate-buffered saline (PBS) and stained with APC-conjugated anti-H-2Kb (MHC class I), FITC-conjugated anti-I-A/I-E (MHC class II), PE-conjugated anti-CD80, and APC-conjugated anti-CD86, along with PE-Cy7-conjugated anti-CD11c (BD Pharmingen) for 30 min at 4 °C. The cells were then washed three times with PBS and resuspended in 500 μl of PBS. Surface markers for BMDC maturation were analyzed by FACSCalibur flow cytometry (BD Biosciences). DCs were stimulated with LPS in medium as a positive control or with medium alone as an untreated control. Cytotoxicity was analyzed by staining for surface-exposed phosphatidylserine with FITC-annexin V in combination with propidium iodine (PI) (BD Biosciences) according to the manufacturer’s instructions.

## Additional Information

**Accession Codes**: Coordinates and structure factors have been submitted to the PDB, accession number 4WZF.

**How to cite this article**: Dong, W. *et al.* Crystal structural basis for Rv0315, an immunostimulatory antigen and inactive beta-1,3-glucanase of *Mycobacterium tuberculosis. Sci. Rep.* **5**, 15073; doi: 10.1038/srep15073 (2015).

## Supplementary Material

Supplementary Information

## Figures and Tables

**Table 1:** Data collection and refinement statistics of the native Rv0315.

	Rv0315
Data collection	
Space group	C121
Cell parameter(a, b, c(Å))	135.13, 65.85, 67.45
α, β, γ	90.00°, 105.17°, 90.00°
Wavelength	0.97917
Resolution range (Å)	24.78–1.70
No. of reflections	62,875
% Completeness	98.4 (99.9)
Rmerge (last shell)	13.6% (22.8%)
I/δ (last shell)	9.5 (7.1)
Redundancy (last shell)	4.0 (4.0)
Refinement	
Resolution (Å)	24.78–1.70
*R*_*work*_/*R*_*free*_	18.4/21.4
No of protein atoms	3857
No. of solvent atoms	681
No. of ions/ligands	14
r.m.s.d.	
Bond length (Å)	0.0070
Bond angle (°)	1.172
B factor (Å^2^)	22
Ramachandran plot: core, allow, disallow	97%, 3%, 0
