# Supplementary material for: Crystal structural basis for Rv0315, an immunostimulatory antigen and inactive beta-1,3-glucanase of Mycobacterium tuberculosis
Source: Sci Rep. 2015 Oct 15;5:15073. doi: 10.1038/srep15073 (PMC4606783; doi:10.1038/srep15073)

Crystal structural basis for Rv0315, an immunostimulatory antigen and pseudo beta-1,3-glucanase of *Mycobacterium tuberculosis*

Wanyu Dong[1,2], Junhua Huang[1,2], Yanan Li[1,2], Yubei Tan[1,2], Zhou Shen[1,2], Yunfeng Song[1,2], Dang Wang[1,2], Shaobo Xiao[1,2], Huanchun Chen[1,2], Zhen F. Fu[1,2,3] * ,Guiqing Peng[1,2,]*

[1] The National Key Laboratory of Agricultural Microbiology, Huazhong Agricultural University, Wuhan, Hubei, China,

[2] College of Veterinary Medicine, Huazhong Agricultural University, Wuhan, Hubei, China,

[3]Departments of Pathology, College of Veterinary Medicine, University of Georgia, Athens, GA 30602, USA.

* To whom correspondence should be addressed: Dr. Guiqing Peng, State-key Laboratory of Agricultural Microbiology, College of Veterinary Medicine, Huazhong Agricultural University, Wuhan, 430070, China. Email: penggq@mail.hzau.edu.cn

Dr. Zhen F. Fu, Department of Pathology, College of Veterinary Medicine, University of Georgia, 501 D.W. Brooks Drive, Athens, GA 30602, USA. Phone: 706-542-7021, Fax: 706-542-5828, E-mail: zhenfu@uga.edu

Supplemental Data

Supplementary Figure Legends

Fig. S1. The amino acid sequence of Rv0315 is compared among different strains of *Mtb*.

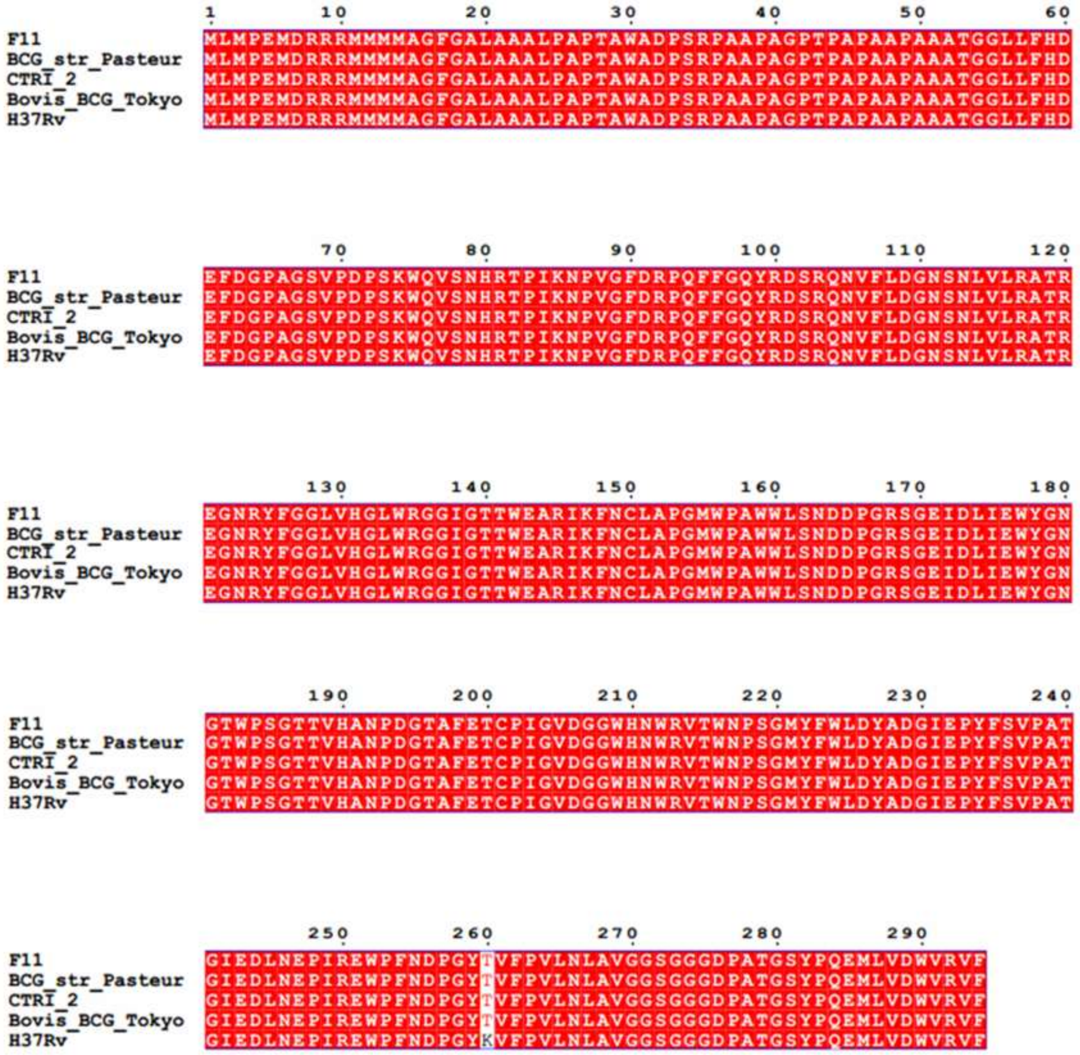

Supplement: Supplementary Information [file srep15073-s1.pdf]
